# Dialyzer Reuse and Outcomes of High Flux Dialysis

**DOI:** 10.1371/journal.pone.0129575

**Published:** 2015-06-09

**Authors:** Christos Argyropoulos, Maria-Eleni Roumelioti, Abdus Sattar, John A. Kellum, Lisa Weissfeld, Mark L. Unruh

**Affiliations:** 1 Department of Internal Medicine, Division of Nephrology, University of New Mexico, Albuqurque, New Mexico, United States of America; 2 Department of Epidemiology and Biostatistics School of Medicine, Case Western Reserve University, Cleveland, Ohio, United States of America; 3 Department of Critical Care Medicine, CRISMA Laboratory, University of Pittsburgh, Pittsburgh, Pennsylvania, United States of America; 4 Department of Biostatistics University of Pittsburgh, Pittsburgh, Pennsylvania, United States of America

## Abstract

**Background:**

The bulk of randomized trial evidence for the expanding use of High Flux (HF) hemodialysis worldwide comes from two randomized controlled trials, one of which (HEMODIALYSIS, HEMO) allowed, while the other (Membrane Outcomes Permeability, MPO) excluded, the reuse of membranes. It is not known whether dialyzer reuse has a differential impact on outcomes with HF vs low flyx (LF) dialyzers.

**Methods:**

Proportional Hazards Models and Joint Models for longitudinal measures and survival outcomes were used in HEMO to analyze the relationship between β2-microglobulin (β2M) concentration, flux, and reuse. Meta-analysis and regression techniques were used to synthesize the evidence for HF dialysis from HEMO and MPO.

**Findings:**

In HEMO, minimally reused (< 6 times) HF dialyzers were associated with a hazard ratio (HR) of 0.67 (95% confidence interval, 95%CI: 0.48–0.92, p = 0.015), 0.64 (95%CI: 0.44 – 0.95, p = 0.03), 0.61 (95%CI: 0.41 – 0.90, p = 0.012), 0.53 (95%CI: 0.28 – 1.02, p = 0.057) relative to minimally reused LF ones for all cause, cardiovascular, cardiac and infectious mortality respectively. These relationships reversed for extensively reused membranes (p for interaction between reuse and flux < 0.001, p = 0.005) for death from all cause and cardiovascular causes, while similar trends were noted for cardiac and infectious mortality (p of interaction between reuse and flux of 0.10 and 0.08 respectively). Reduction of β2M explained only 1/3 of the effect of minimally reused HF dialyzers on all cause mortality, while non-β2M related factors explained the apparent attenuation of the benefit with more extensively reused dialyzers. Meta-regression of HEMO and MPO estimated an adjusted HR of 0.63 (95% CI: 0.51–0.78) for non-reused HF dialyzers compared with non-reused LF membranes.

**Conclusions:**

This secondary analysis and synthesis of two large hemodialysis trials supports the widespread use of HF dialyzers in clinical hemodialysis over the last decade. A mechanistic understanding of the effects of HF dialysis and the reuse process on dialyzers may suggest novel biomarkers for uremic toxicity and may accelerate membrane technology innovations that will improve patient outcomes.

## Introduction

Patients with end-stage renal disease (ESRD) undergoing maintenance hemodialysis (HD) have a high mortality rate despite continuous improvements in technology and dialytic care [1]. Worldwide 60% of patients are dialyzed with High Flux (HF) dialyzers. These dialyzers facilitate the removal of toxins larger than urea [2], based on observational and epidemiologic studies [3–7] suggesting a clinical benefit from their use. Nevertheless, a Cochrane meta-analysis of ten randomized controlled trials (RCTs) on 2915 patients has shown a benefit of HF dialysis on cardiovascular mortality but not on all-cause mortality [8]. In this meta-analysis, the majority of patients (85%), events (96.5%) and thus, the overwhelming evidential weight (96.1%) for or against the benefits of HF dialysis comes from two RCTs; i.e. HEMODIALYSIS, (HEMO) [8] and the Membrane Permeability Outcome, (MPO) [9]; which yielded numerically different estimates for the effect of HF membranes on survival.

Dialyzer reuse, was permitted only in HEMO [9,10] and is considered a key interventional difference between the two RCTs of HF dialysis [11,12]. Therefore reuse has been offered as one potential explanation for the somewhat divergent findings of these two studies. Nevertheless, an assessment of the impact of reuse on outcomes of HF dialysis in HEMO has not been performed to date. Such an assessment may inform technology use decisions worldwide, as HF membranes are the de-facto dialysis industry standard. Reuse of HF membranes is still practiced in the US [13] and is widely employed in resource limited settings [14,15] providing a justification for revisiting the effects of reuse on outcomes in HEMO

The major objective of this work is to explore the associations of non-reused and minimally reused HF v.s. LF dialyzers in the context of HEMO and MPO. Secondarily we were interested to assess these effects in the medically important subgroups of patients with hypo-albuminemia or diabetes.To describe the relative role of β2-microglobulin (β2M) concentration, membrane flux and reuse on patient outcomes in HEMO, we supplemented conventional survival analyses with emerging techniques for the simultaneous (joint) modeling of patient level biomarkers and survival. Meta-regression techniques were used to assess the impact of non-reused HF dialyzers simultaneously considering the evidence in both HEMO and MPO. Finally, we undertook simulations of patient survival under various scenarios of reuse and adoption of HF dialysis.

## Subjects and Methods

### Subjects and Data

We used the patient level data from the HEMO RCT of the effects of HD dose and membrane flux [16] as distributed by the National Institute of Diabetes and Digestive and Kidney Diseases (NIDDK). Data are available from the NIDDK repository website http://www.niddkrepository.org. We classified HEMO participants into cohorts of increasing reuse, based on monthly collected dialysis information. Patients were assigned to the most commonly observed reuse method in their study records since the same reuse technique was utilized in over 92% of the sessions for the vast majority (85%) of the study patients. Patients were classified as dialyzed with non-reused membranes, if the number of reuses for their dialyzer was zero for every monthly dialysis session in the study records. We used a two-step procedure to classify all other patients to cohorts of increasing dialyzer reuse. At the first step we computed the 97.5th quartile for the number of reuses for each study participant during the entire study period. Subsequently, these individual measures of reuse exposure were grouped into quartiles. In addition, the cumulative mean number of filter reuses was computed on a monthly basis for each patient and was used for sensitivity analyses in survival and Joint Models. We carried out this sensitivity analysis to ensure that the results were robust to our approach of assigning reuse. β2M clearance was calculated with a variable-volume single-pool model, adjusted for the fluid removal during dialysis [17,18].

The Institutional Review Board (IRB) of the University of New Mexico approved this secondary analysis of the HEMO Randomized Controlled Trial (Study ID 13–468 decision of 12/12/2013). All study participants had provided informed consent to participate in HEMO, and the ethics committees/IRBs of participating centers had reviewed and approved the consent form during protocol review. These documents and the HEMO can be downloaded from the NIDDK repository. Individual HEMO participants were not consented for this secondary analysis, because the data as distributed by the NIDDK has been de-identified. Furthermore the data use agreement between the investigators of this paper and NIDDK prohibits us from making any contact to identify individuals, families or communities. The IRB of the University of New Mexico waived the requirement for an informed consent for this secondary analysis after reviewing the original consent form that HEMO participants signed upon their enrollment, the data use agreement between the investigators and NIDDK and the associated research protocol submitted to the NIDDK.

### Statistical Methods

#### Survival Modeling

We applied the proportional hazard (Cox) model to compute hazard ratios (HRs) in patient cohorts. These models were adjusted for all *baseline* covariates used in the initial trial report: Kt/V arm assignment, age, gender, presence of diabetes, race, co-morbidity score (ICED), duration of ESRD, serum albumin level, dialysis dose (Kt/V arm assignment) and flux. We also adjusted survival models for vascular access and residual urine output in order to minimize residual confounding from omitting these prognostically important variables. The role of dialysis access is well established as a predictor of survival in the nephrology literature (e.g. in the USRDS US registry[19], the Renal Disease Registry in Ontario[20] and the international comparisons in the DOPPS registry[21]). We have previously found access to be a confounder in statistical analysis of the CHOICE prospective dialysis cohort in the US[22], while more recently access was found to modify the risk associated with dialysis in for-profit v.s. non-for-profit dialysis units in the US[23]. Residual renal function (RRF) has been found to be a predictor of survival in studies from the Netherlands (NECOSAD-2)[24], in the US[25] and even in the context of incremental dialysis[26]. It is a topic of re-emerging importance in the dialysis literature (see [27]for an excellent review). To account for clustering of dialysis patients in units, we computed robust (sandwich) standard error estimates in all Cox models. This methodology[28], has been become the de facto standard for analyzing hemodialysis outcomes (see[29–33] for indicative, non-exhaustive list of references) since its initial introduction. Patient’s follow-up time was censored if they were alive at the end of the study or when they received a kidney transplant, but not when they dropped out from the study in accordance with the HEMO statistical analysis plan [9].

#### Repeated Measures Modeling

We related β_2_M *clearance* to the extent of reuse with mixed effect models that employed a nested, individual-within-center random effect structure for the correlation between repeated measures obtained in the same individual. Generalized Estimating Equations (GEEs) for binary outcomes were used to analyze the membrane material (polysulfone vs. non-polysulfone). The use of the two different repeated measures methodologies is justified by the design of the HEMO study and our research questions. In particular, the HEMO protocol tracked clearance of beta 2 microglobulin at the patient level. Therefore to analyze the relevant variables (beta 2 microglobulin and clearance) an individual level approach such as a (generalized) linear mixed model is indicated. On the other hand, HEMO did not enforce the use of specific membrane materials on a patient or a study center level, so that analyzing the population exposures becomes the relevant question. GEEs implement population level models, and are the appropriate methodology to use for our research question[34].

#### Joint Modeling of predialysis β2M concentration, and survival

**Fig 1:**
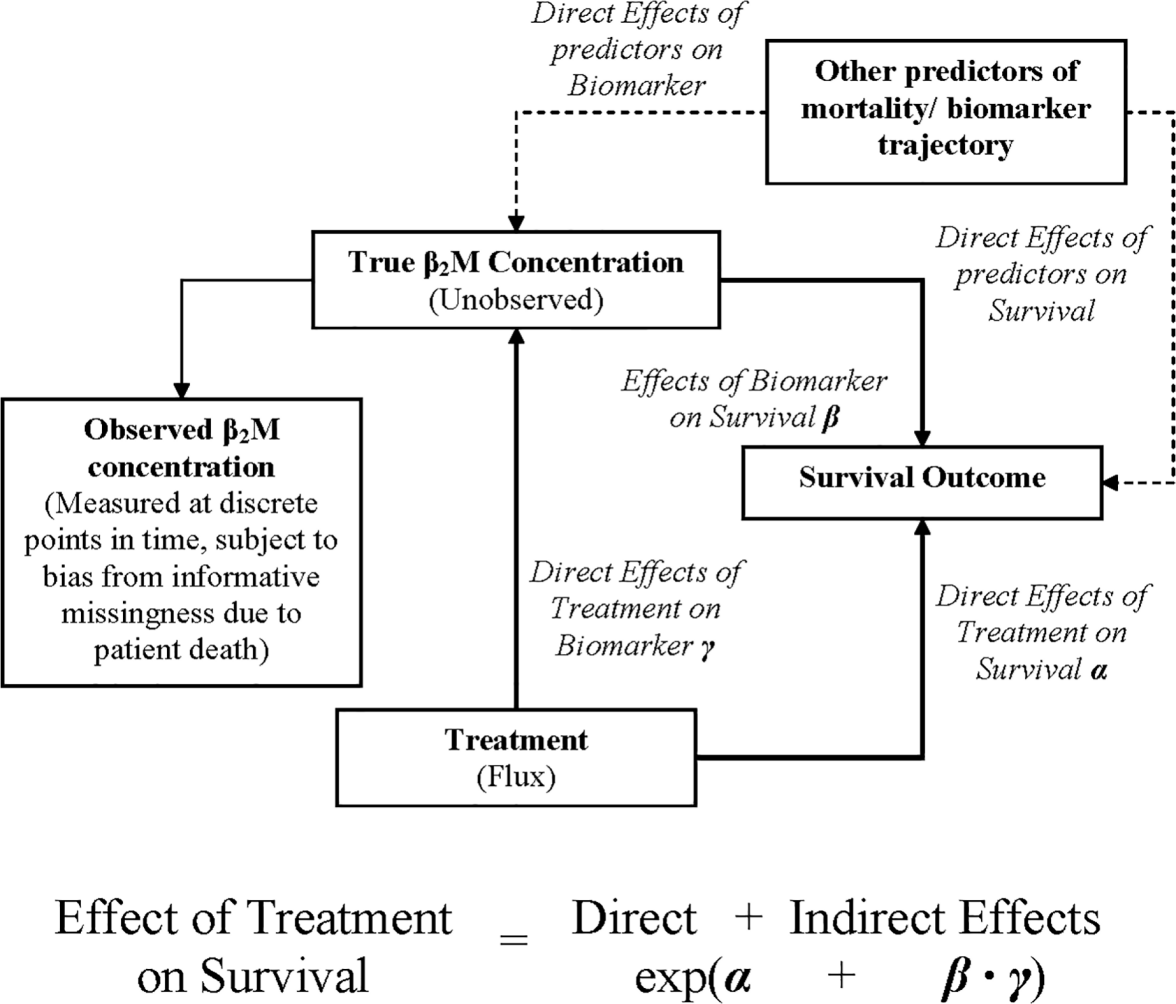
Conceptual depiction of the Joint Modeling Framework. A joint model is an analytical method for the simultaneous estimation of treatment effects on survival outcomes, the effects of treatment on the concentration of a biomarker over time (trajectory function) and the effects of the latter on survival. As the true biomarker level is unobserved, a JM has to reconstruct its trajectory before these effects can be estimated. Other factors may affect survival either directly or indirectly (through the biomarker concentration) and they can be used to adjust a JM and thus reduce residual confounding. One possible interpretation of the fitted joint model effects on survival assigns *direct* treatment effects (not associated with changes in the biomarker trajectory, α) and *indirect* treatment effects (associated with changes in the biomarker concentration, given by the product of β and γ). Hence, the overall treatment effect in the Hazard Ratio scale is the (exponentiated) sum of direct and indirect effects. This effect is usually numerically similar to the one estimated by a Cox model.

We characterized the relationship between flux, β2M concentration, and survival using a *joint model* (JM) [35] for repeated measures and survival. A JM is an analytical method for the simultaneous estimation of treatment effects on survival outcomes, the effects of treatment on the concentration of a biomarker over time (trajectory function) and the independent effects of the latter on survival. Since other factors may affect survival either directly or indirectly (through the biomarker concentration) the JM may adjust for them to reduce residual confounding. The JM overcomes the biases implied by the simplistic inclusion of time updated values of biomarkers in survival models. These biases which arise from measurement error and informative censoring (e.g. lack of measurements after patient’s death) may seriously compromise the inferences of the survival models[36,37]. The interpretation of the fitted joint model assigns *direct* treatment effects (not associated with changes in the biomarker i.e. β2M trajectory and *indirect* treatment effects (associated with changes in the biomarker concentration and the effects of the latter on survival). The overall treatment effect (measured in the log-hazard scale) is the sum of the direct and indirect effects (Fig 1) [38–40] and is usually not different from the one estimated by the Cox model.

To describe the trajectory of β2M over time in HEMO we used a natural cubic spline with internal knots at 45 and 90 days and two external knots at 0 days (beginning of the study) and 6 months after randomization. The adopted specification allowed flexible modeling of a) the non-linear changes in β2M concentration, resulting from changing membrane flux in the six months post-randomization, followed by a b) linear trend afterwards. The justification for this bi-phasic model was based on previous clinical investigations [41–45] and computer simulations [46–48], demonstrating that changes in serum β2M concentration occur within a few weeks after abrupt changes in dialytic clearance with very little change thereafter. As patients in HEMO could be dialyzed either with LF or HF before randomization, we allowed the trajectory of β2M concentration to differ according to both pre-randomization and post-randomization membrane flux. Hence our model anticipated increases or decreases in β2M concentration (e.g. patients switching from HF to LF membranes and vice versa) and stable β2M trajectories (e.g. patients who were randomly assigned to the same flux as the filters used prior to study enrollment).

The selection of the position of the internal knots was motivated by the sampling frequency of predialysis β2M concentration in HEMO: β2M was measured on the first and second month and then every other month (HF arm) and at months 1, 4 and yearly thereafter (LF arm). Hence, we specified the position of the internal knots to ensure that sufficient data points were available at each interval (0–45 days, 45–90 days, 90–180 days and 180 days-end of follow up) for the estimation of the spline coefficients. Of note, only a small number of HEMO study participants (<10%) had β2M determinations at baseline and these measurements were included in the dataset that was supplied to the JM.

We utilized the capability of JMs to yield dynamic, subject specific survival probabilities from a baseline set of measurements [31] in order to predict the expected outcomes (difference in 5 year survival probability) under all possible flux and reuse combinations that were utilized in HEMO (S1 Text: Simulations). These simulations allow us to predict the effects of reusing either LF or HF dialyzers in a cohort of patients with similar characteristics to the HEMO study participants. These are Bayesian probability estimates which do not admit a conventional p-value calculation for their comparison. Hence, we adopted the literature convention, and considered differences in survival as “significant” if the corresponding 95% Credible Interval (CrI, the range of values in which 95% of the probability is contained) excludes zero. Further technical details about the JM are given in S1 Text.

#### Evidence Synthesis of HEMO and MPO

HEMO and MPO are the largest prospective RCTs of membrane flux, accounting for >96% of the total weight of evidence in a recent Cochrane Group Systematic Review of the impact of HF dialyzers on outcomes [8]. The aforementioned meta-analysis did not account for differences in reuse, patient demographics, dialysis dose or statistical adjustments for baseline patient characteristics that were employed in HEMO and MPO (S1 Text). To account for the differences in interventions, patients in HEMO were cross-classified according to the single-pool Kt/V (spKt/V) arm(coded as standard or high), flux and quartiles of reuse, before computing discrete outcomes (number of events and patient years) for *unadjusted meta-analyses*. Since the average dialysis dose employed in MPO was similar to the standard dose employed in HEMO, all patients from MPO were classified as receiving standard dialysis with either LF or HF membranes. To *align the statistical models used in the trials*, we re-analyzed survival in HEMO using the same statistical model that the MPO investigators used in their report. Investigators in MPO, adjusted the hazard ratio of flux for age, Kt/V, gender, diabetes, comorbidity score and type of access. This information was extracted from the MPO publication (Table 4 in [19]). We applied the same model in HEMO to yield adjusted estimates for high flux dialysis within each of the eight cohorts defined on the basis of reuse quartile and Kt/V assignment. To combine the adjusted hazard ratios from both studies, we utilized random effects meta-regression. Meta-regression adjusted these flux estimates for spKt/V and the average reuse number of the patients in each HEMO reuse quartile. As dialyzers were not reused in MPO, the corresponding reuse number was set to zero.

We also undertook subgroup analysis in hypoalbuminemic patients (a prespecified subgroup in the MPO analytic protocol). Secondary analyses of the MPO and other observational datasets [6] suggest that diabetic patients may derive an additional benefit from HF dialysis. Consequently, we also considered a subgroup analysis of the diabetic study participants in the two studies. Further technical details about the meta-analysis are given in Supplementary Methods (S1 Text, Section: “Meta-analysis and Meta-regression of HEMO and MPO”).

All statistical analyses were performed with R versions 2.9.2–2.15.1; joint modeling was undertaken with the R package “JM” version 1.0–1.1 [34,35].

## Results

### Patients and Reuse Exposures

Characteristics of patients were similar across quartiles of reuse with the exception of patients of white race and female gender who were more likely to be represented in the lowest reuse quartile (Table 1). As a group, patients at the lowest reuse quartile were also more likely to be enrolled later in the trial, to be dialyzed with HF dialyzers before randomization and to have residual urine output (Table 1). However, within reuse quartiles there were no substantial differences in characteristics in patients randomized to LF versus HF membranes (S1–S4 Tables). There was no statistically significant difference in the odds of using polysulfone vs. non-polysulfone dialyzers between the two flux arms in the first (Odds Ratio (OR): 0.88, 95%CI: 0.60–1.27), p = 0.48) and across the remaining reuse quartiles (p for the interaction between flux and 2^nd^ -4^th^ quartiles: 0.21, 0.66, 0.27). The most common reuse method involved peracetic acid (PAA, 45.3% of patients), followed by formaldehyde (FAH, 25.1%), gluteraldehyde (GAH, 10.7%) and heated citric acid (HCA, 9.4%); only a minority (9.5%) of all HEMO patients were dialyzed with non-reused filters.

**Table 1:** Baseline Characteristics of HEMO Participants by Reuse Quartile.

	Reuse Quartile				p
**N**	531	443	448	404	
**Range of Number of Reuses**	0–6	7–12	13–17	>17	
**Flux Assignment**					0.59
High Flux	48% (254)	48% (213)	52% (234)	52% (209)	
**Kt/V Assignment**					0.27
High Kt/V	50% (268)	53%(236)	48%(216)	47%(190)	
**Prerandomization Membrane Flux**					<0.001
High Flux	47% (246)	66% (289)	59% (264)	72% (289)	
**Age**	58 ± 14	58 ± 14	58 ± 13	57 ± 15	0.90
**Race**					<0.001
Black	56% (300)	72% (321)	62% (279)	60% (244)	
**Diabetic Status**					0.52
Diabetic	44% (234)	46% (205)	46% (207)	42% (169)	
**Gender**					0.33
Female	59% (312)	56% (248)	53% (237)	56% (228)	0.045
**Duration (years)**	3.43 ± 4.17	3.86 ± 4.44	4.06 ± 4.75	3.71 ± 4.05	0.56
**Cause of ESRD**					
Glomerulonephritis	15% (79)	15% (66)	13% (60)	12% (47)	
Hypertension	28% (150)	32% (140)	32% (143)	36% (146)	
Diabetes	37% (199)	38% (167)	39% (176)	34% (139)	
Ischemic Nephropathy	7% (37)	5% (21)	6% (25)	4% (18)	
Acute Renal Disease	4% (22)	3% (12)	1% (6)	2% (10)	
Other	9% (44)	7% (37)	9% (38)	12% (44)	
**Albumin (g/dl)**	3.61 ± 0.35	3.63 ± 0.35	3.63 ± 0.37	3.63 ± 0.36	0.66
Residual Renal Function*					0.007
Urine Volume (>200 ml/day)	17% (90)	9% (41)	14% (63)	13% (54)	
**ICED**	1.93 ± 0.84	1.98 ± 0.83	1.93 ± 0.83	2.06 ± 0.82	0.085
**Vascular Access**					
Arteriovenous Fistula	35% (188)	33% (148)	34% (151)	31% (126)	0.27
Arteriovenous Graft	57% (305)	58% (258)	61% (274)	61% (247)	
Catheter/Other	7% (38)	8% (37)	5% (23)	7% (31)	
**Year of Randomization**(since the beginning of HEMO)	3.19 ± 1.65	1.69 ± 1.41	1.62 ± 1.53	1.92 ± 1.68	<0.001

Summary statistics are presented as mean ± standard deviation and as % (frequencies) for continuous and categorical variables respectively. Tests used to compare variables Pearson test (categorical)^,^ Kruskal-Wallis (continuous). The total number of patients (1826) differs from the number of patients enrolled in HEMO, because there was no available information about reuse for 20 subjects who died before their first post-enrolment evaluation. ICED: Index of Coexistent Diseases.

The method (S5 Table) and extent of reuse differed among different dialyzers, ranging from 10.9 ± 5.6 (PAA) to 15.2 ± 5.0 (FAH) times in accordance with the labeling information of the dialyzers and dialysis center practices. Mixed model analysis demonstrated wide differences in the average clearance of reused HF dialyzers by reuse method: 2.72±1.13 ml/min (FAH, p = 0.016), -1.26±1.31 ml/min (GAH, p = 0.33), 5.71±1.35 ml/min (HCA, p<0.001), -12.9±1.1 ml/min (PAA, p<0.001), relative to non-reused ones (38.4±0.97 ml/min).

The average predialysis β2M concentration in patients dialyzing with non-reused HF membranes was 31.1 **±** 0.99 mg/l; β2M was non-significantly increased by FHA, GAH or HCA reprocessing: 1.57 **±** 1.15 mg/l (p = 0.17), 1.59 **±** 1.39 mg/l (p = 0.23), 1.54 **±** 1.40 mg/l (p = 0.27) respectively. Only reprocessing with PAA was associated with a moderately higher β2M concentration of 4.03 β2M **±** 1.08 mg/l (p<0.001) relative to non-reused HF dialysis in these unadjusted analyses. After adjusting for a large number of covariates (Kt/V, race, gender, diabetes, comorbidity score, duration of dialysis dependency, vascular access, transplantation status, smoking, BMI, albumin, urine output (as more or less than a cup per day) and study center, reuse with PAA was not associated with higher β2M concentration (difference of -0.28 ± 1.65, p = 0.87).

**Fig 2:**
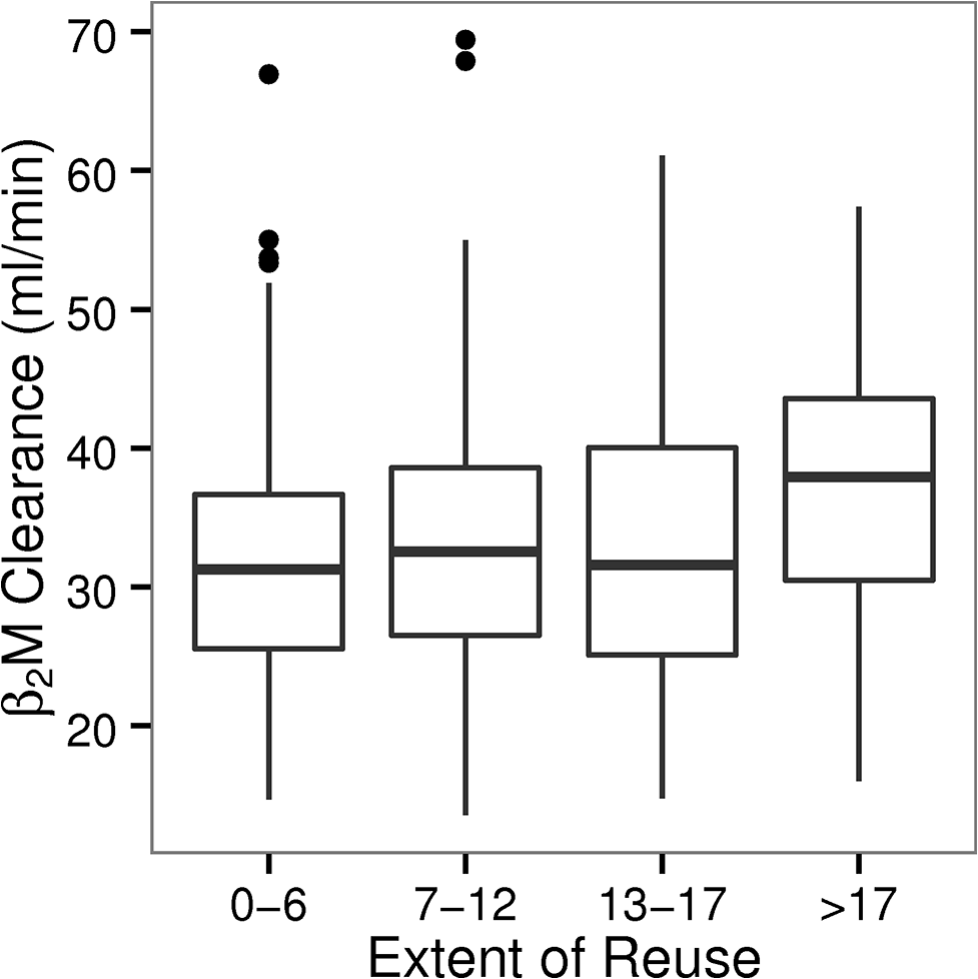
Distribution of predicted β2M clearance across quartiles of membrane reuse in HEMO. Controlling the extent of reuse by the HEMO study protocol yielded average clearances that were equivalent across reuse quartiles in patients receiving HF dialysis. Average β2M clearance by HF dialyzers in the 2nd to 4th reuse quartile, was 1.26±1.02 (p = 0.21), 1.47±1.00 (p = 0.14) and 5.41±1.03 (p<0.001) ml/min higher relative to the first quartile (31.8±0.69 ml/min).

In summary, the potentially large impact of reuse method on β2M clearance did not translate into a substantial effect on predialysis β2M concentration. As the HEMO study protocol controlled the maximum number of reuses, according to the effects of reuse protocol on clearance, the average β2M clearance did not materially differ across reuse quartiles (Fig 2).

### Relative effect of HF dialysis on survival depends on the extent of dialyzer reuse

The effects of HF dialysis were modified by the extent of dialyzer reuse. In adjusted Cox models (S6 Table), the interaction between flux and reuse was statistically significant for all-cause (p<0.001) and cardiovascular (p = 0.005) mortality. A trend for higher relative mortality for cardiac and infectious causes (p = 0.10 and p = 0.08 for the interaction between HF and reuse) was also noticed. These statistical interactions imply that a beneficial effect of HF *relative to LF* dialysis on all-cause and cause-specific mortality was attenuated for patients dialyzed with filters that were reused more than 6 times (Table 2). Sensitivity analyses, which used the time updated (cumulative mean) number or reuses did not yield substantially different HR estimates (S7 Table). In analyses adjusting for reuse method, the interaction between flux and extent of reuse remained significant. In these analyses, FAH and GAH were associated with numerically worse relative outcomes to other reuse methods (not shown). There was no interaction between reuse, baseline urine production and dialysis flux (p for all three way interactions > 0.77) suggesting that the effects of reuse and flux did not differ between anuric patients and those with minimal RRF.

**Table 2:** Hazard Ratio of Death for All Cause and Cause Specific mortality between High Flux and Low Flux Dialysis versus extent of Reuse.

	1^st^ Quartile (N = 526)	2^nd^ Quartile (N = 436)	3^rd^ Quartile (N = 438)	4^th^ Quartile (N = 403)
*Number of Reuses*	0–6	6–12	12–17	>17
**All Cause Mortality**	0.67 (0.48–0.92) p = 0.015	0.86 (0.66–1.12) p = 0.27	0.86 (0.64–1.14) p = 0.29	1.45 (1.13–1.86) p = 0.003
**Cardiac Mortality**	0.64 (0.44–0.95) p = 0.03	0.69 (0.44–1.08) p = 0.10	0.83 (0.55–1.26) p = 0.37	0.98 (0.68–1.41) p = 0.91
**Cardiovascular Mortality**	0.61 (0.41–0.90) p = 0.012	0.62 (0.40–0.95) p = 0.029	0.81 (0.55–1.20) p = 0.30	1.16 (0.88–1.54) p = 0.29
**Infectious Mortality**	0.53 (0.28–1.02) p = 0.057	1.11 (0.69–1.81) p = 0.66	0.64 (0.35–1.18) p = 0.16	1.30 (0.85–1.99) p = 0.22

Hazard Ratio (HR) estimates were obtained from a Cox regression model adjusting for reuse quartile, flux and Kt/V assignments, age, sex, diabetes, duration of ESRD dependency (upon study enrolment), ICED, albumin, vascular access, pre-randomization flux, residual urine volume, and finally the interaction between reuse quartile and flux; the baseline hazard was stratified by study centre. Reported Relative Risks are model predictions and associated 95% CI for patients dialyzed with high flux relative to patients dialyzed with low flux, reused to the same extent as the former. Results based on 1803 patients with complete data for model fitting.

When interactions between albumin concentration at baseline and HF dialysis were examined, we noted a statistically significant effect for cardiac (p = 0.009), and a trend for cardiovascular and all-cause mortality (p = 0.051 and p = 0.096 respectively). The direction of the interaction term indicates a larger benefit of HF dialysis for patients with lower albumin levels. No interaction was noted between the presence of diabetes at baseline and all-cause or cause specific mortality (p>0.20 for all interactions).

To better understand these findings we undertook a number of joint modeling analyses aiming to ascertain whether reuse a) affected the exposure to middle molecules (β2M concentration) b) whether the association of a given β2M concentration with survival differed according to the extent of dialyzer reuse and c) whether the apparent attenuation of the protective effect of HF dialysis reflected worse outcomes with reused high flux membranes or improved survival in patients exposed to reused LF dialyzers.

### The effects of reuse on outcomes of HF dialysis are not related to middle molecules

#### Dialyzer reuse is not associated with differential changes in β2M concentration

**Fig 3:**
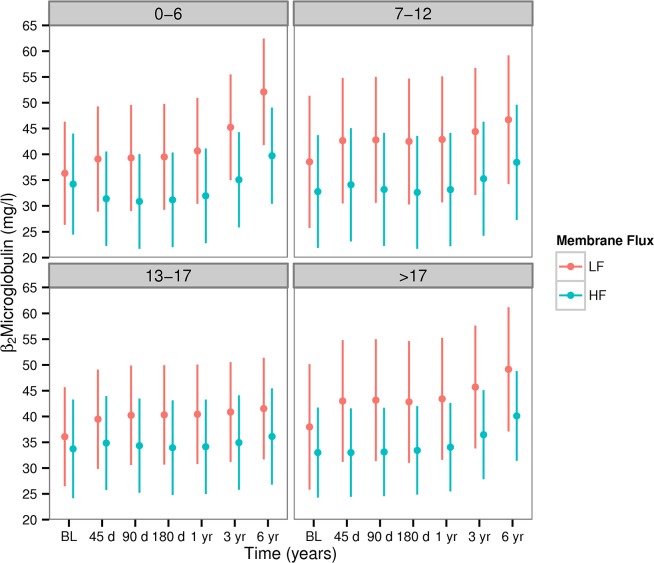
Predicted predialysis β2M concentrations across reuse quartiles and membrane flux in HEMO participants. These predictions were obtained by fitting a Joint Model to the HEMO dataset which was then used to predict the β2M concentration for the HEMO study participants at different time points. The panels show the mean and standard deviation obtained by averaging individual level predictions at each time point. Separation between the high (HF) and the low flux(LF) arms in terms of the β2M concentration were established early in the trial. There was very little difference in the separation of flux arms across the quartiles of reuse, i.e. estimated difference (SE) in β2M concentration between HF and LF was -8.27 (0.56), -9.67 (1.22), -6.72 (1.06), and -8.42 (1.26) mg/L in the 1st-4th reuse quartiles respectively.

In the first six months of HEMO, β2M increased in patients assigned to LF membranes and decreased in patients assigned to the HF arm (Table 3, acute slope); the rate of change of β2M was smaller after the first six months (Table 3, chronic slope). The rate of accumulation of β2M in patients exposed to reused LF membranes was smaller by 1.01 to 2.01 mg/l/year compared to the non-reused membranes. Rates of accumulation of β2M did not differ between HF and LF membranes subjected to the same extent of reuse (p: 011–0.98 Table 3). Differences in the concentration of β2M between the HF and LF arms were established early and maintained throughout the duration of the study (Fig 3). Adjusted mean differences (SE) in predialysis β2M concentration between LF and HF dialysis were not materially different in the various reuse quartiles: -8.29(0.99), -9.68(1.22), -6.73(1.06), -8.42(1.27) mg/dl for 0–6, 7–12, 12–17, >17 reuses respectively.

**Table 3:** Average Acute (first six months) and Chronic Slopes (> 6 months) of Predialysis β2M Concentration according to Reuse Quartile and Membrane Flux.

	Acute Slope (0–6 months)			Chronic Slope (>6 months)		
*Reuse*	*Estimate (SE)*	*Pǂ*	P*	*Estimate (SE)*	*Pǂ*	P*
**Low Flux**						
0–6	6.61 (1.77)	Ref	Ref	2.27 (0.37)	Ref	Ref
7–12	-1.49 (2.71)	0.58	Ref	-1.56 (0.49)	0.002	Ref
13–17	1.50 (2.65)	0.57	Ref	-2.01 (0.49)	<0.001	Ref
>17	0.46 (2.94)	0.89	Ref	-1.07 (0.50)	0.003	Ref
**High Flux**						
0–6	-5.44 (1.77)	Ref	<0.001	1.54 (0.28)	Ref	0.11
7–12	4.66 (2.67)	0.081	0.004	-0.51 (0.38)	0.17	0.49
13–17	5.00 (2.52)	0.047	0.001	-1.26 (0.37)	<0.001	0.98
>17	5.06 (2.85)	0.076	0.021	-0.45 (0.43)	0.28	0.78

Estimates of slopes are given in Δmg/L β2M/year relative to the first quartile of reuse in each flux arm (entry in rows labelled “0–6”); all estimates were obtained by a joint model for the (untransformed) β2M concentration and survival. The longitudinal and survival submodels adjusted for reuse quartile, flux and Kt/V assignments, age, sex, diabetes, duration of ESRD dependency (upon study enrolment), ICED, albumin, vascular access, pre-randomization flux, residual urine volume, study center and the interaction between reuse quartile by flux. To model the changing trajectories of β2M as a result of patients being assigned to dialyzers with a different flux than the one used before randomization, the longitudinal component also included interactions between pre- and post-randomization flux and a spline function of time since randomization. The baseline hazard of the survival submodel was modeled with a natural spline with two internal knots at 45 and 90 days and two boundary (external) knots at 0 and 180 days.

ǂP-values comparing each slope to the reference category within each flux arm * P value comparing the slope between the same reuse category of the two flux arms.

#### Changes in β2M account only partly for the effects of non-reused HF dialyzers

In joint models, serum β2M concentration was a statistically significant predictor of survival: each 10 mg/L elevation was associated with an estimated HR of 1.16 (95% CI: 1.07–1.25, p<0.001). Indirect effects of HF vs. LF dialysis (i.e. those associated with changes in serum β2M) were not different across reuse quartiles (Table 4) and were moderate in magnitude (hazard ratios ranging from 0.87–0.91). On the other hand, direct effects, i.e. not explained by changes in predialysis β2M, differed across reuse quartiles and in fact reversed direction for highly reused membranes (Table 4). Total effects of flux were nearly identical to the results of the Cox models in Table 2 (not shown).

Similar findings were obtained when reuse was treated as a time updated variable in sensitivity analyses: the best fitting JM suggested a quadratic relationship between the direct and total effects of flux and HR of death so that the hazard ratio was increased in patients receiving HF dialysis when these dialyzers were reused more than 17 times (S1 Fig). In sensitivity analyses, the attained β2M concentration did not have a differential association with survival in patients treated with reused HF dialyzers (p for the interaction between indirect effect and flux, indirect effect and cumulative reuse and flux by cumulative reuse: 0.28, 0.10 and 0.25 respectively).

**Table 4:** Joint Model estimates of Adjusted Hazard Ratios for All Cause Mortality associated with the use of High Flux vs. Low Flux Dialyzers under different extents of Reuse.

	Indirect Effect			Direct Effect		
*Reuse*	*Estimate*	*95% CI*	*P*	*Estimate*	*95% CI*	*P*
0–6	0.89	0.82–0.95	<0.001	0.78	0.57–1.06	0.12
7–12	0.87	0.80–0.93	<0.001	0.98	0.75–1.28	0.88
13–17	0.91	0.85–0.96	<0.001	0.98	0.75–1.28	0.87
>17	0.88	0.82–0.95	<0.001	1.72	1.28–2.32	<0.001

Hazard Ratio (HR) estimates were obtained from a Joint Model for pre-dialysis β2M (untransformed) and all cause-mortality as detailed in the methods and the legend of Table 3. Reported Hazard Ratios and associated 95% CI for patients dialyzed with HF are relative to patients dialyzed with LF ones, reused to the same extent as the HF ones.

#### Reuse is not associated with worse outcomes in HF dialysis, but improves outcomes in patients exposed to LF membranes

**Fig 4:**
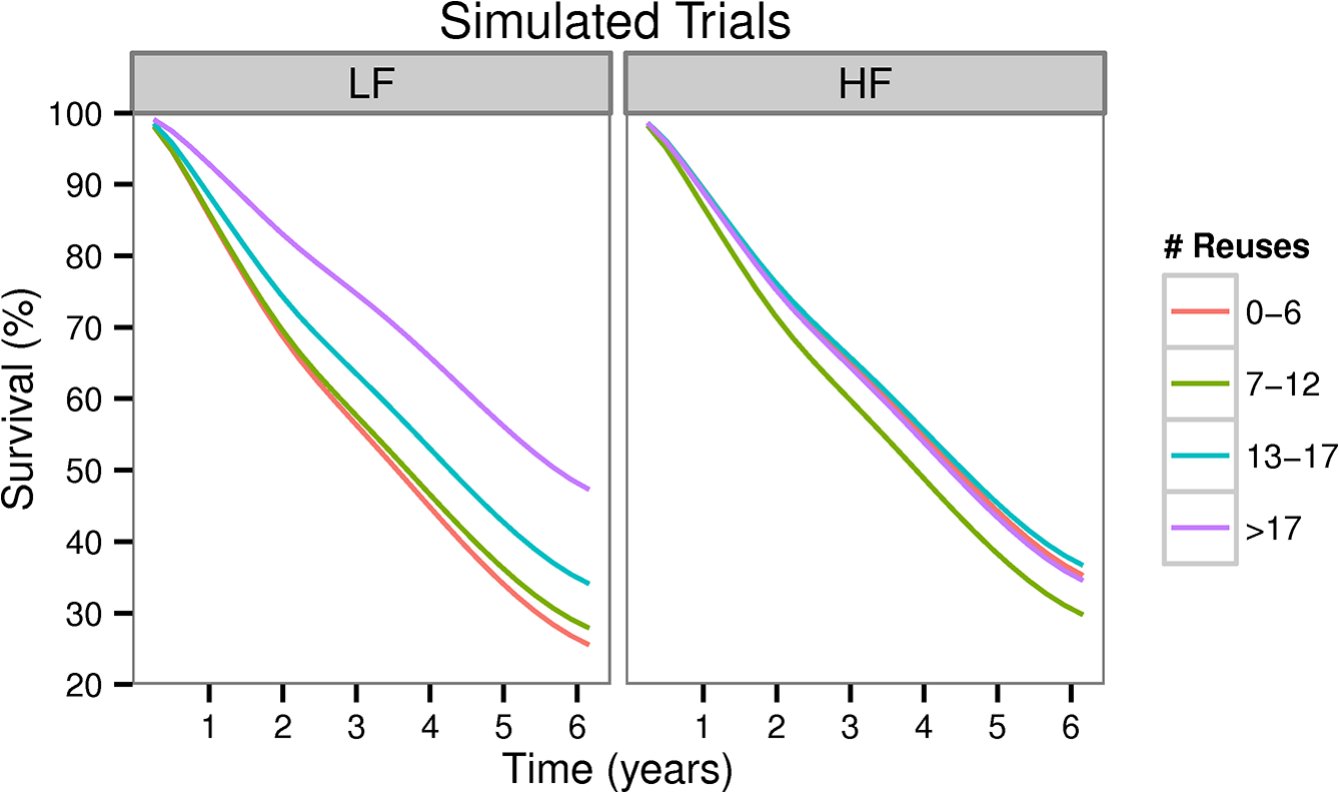
Predicted survival curves obtained by dynamic simulations of Joint Models. These were derived by counterfactually exposing HEMO study participants to all possible flux and reuse combinations. Individual survival probability curves were averaged at each observation point and these population averaged curves are depicted separately for low flux(LF) and high flux(HF) dialysis. Five year survival probabilities (P5, estimate and 95% Credible Interval) were as follows: 33.8% (28.0%-39.4%), 36.1% (30.8%-41.0%), 42.4% (36.9%-47.8%) and 55.6% (49.3%-55.8%) for increasing reuse of LF membranes. P5 (estimates and 95% Credible Intervals) for increasing reuse of HF dialyzers were: 43.7% (37.4%-49.1%), 38.% (32.9%-43.5%), 44.8% (38.1%-50.7%) and 43.0% (37.0%-49.1%).

We used JMs to predict *absolute* survival in a cohort of simulated patients who were exposed to the 8 different combinations of flux and quartiles of reuse. Irrespective of the extent of their reuse, HF membranes are predicted to confer a survival advantage over minimally reused LF dialyzers, e.g. a 9.9% increase in 5 year survival (95% CrI: 1.6%-18.0%) and 9.2% (95% CrI: 1.3–19.4%) for the lowest and highest reuse quartiles respectively. However, compared to minimal reuse of HF dialyzers, extensive reuse of these membranes was not associated with worsening survival, i.e a difference in five year survival probability of -0.7% (95% CrI -10.5% to 6.2% for the highest reuse quartile vs. minimal reuse). The neutral effect of reuse on outcomes of HF dialysis was also evident in the Kaplan Meier curves which were nearly superimposable (Fig 4). On the other hand, reuse of LF membranes modified survival (Fig 4) especially; the highest five year survival probability was noted for the most extensively reused LF dialyzers: 55.6% (95% CrI: 49.3–61.4%).

From a comparative effectiveness perspective, these simulations imply that the effects of HF dialysis in a given reuse setting would depend on the extent of the reuse and the referent (LF vs. HF). In HEMO, reuse of low flux dialyzers was associated with improved absolute clinical outcomes in the LF arm, but was not associated with either worse or improved outcomes in the HF arm. These patterns underlie the apparent attenuation of the hazard ratio of high flux dialysis with reuse (Table 2)

#### Synthesis of HEMO and MPO results suggests a benefit for non-reused/minimally reused HF dialyzers that may be larger in hypoalbuminemic patients

When synthesizing the evidence from the *adjusted analyses* of HEMO and MPO without accounting for the extent of reuse or small molecule clearance, we obtained results compatible with the ones reported in the *unadjusted meta-analysis* by the Cochrane group [8]: HR for all cause mortality of 0.89, 95% CI (0.71–1.11, p = 0.28, S2 Fig panel A) vs. 0.95 (95% CI: 0.87–1.04, p = 0.23[32]). In meta-analysis of adjusted HRs, HF dialysis was associated with a statistically significant effect in the subgroup of hypoalbuminemic patients (HR: 0.70, 95%CI: 0.52–0.93, p = 0.013, S2 Fig, panel B) but not those with diabetes (HR: 0.87, 95%CI: 0.68–1.11, p = 0.26, S2 Fig, panel C).

**Fig 5:**
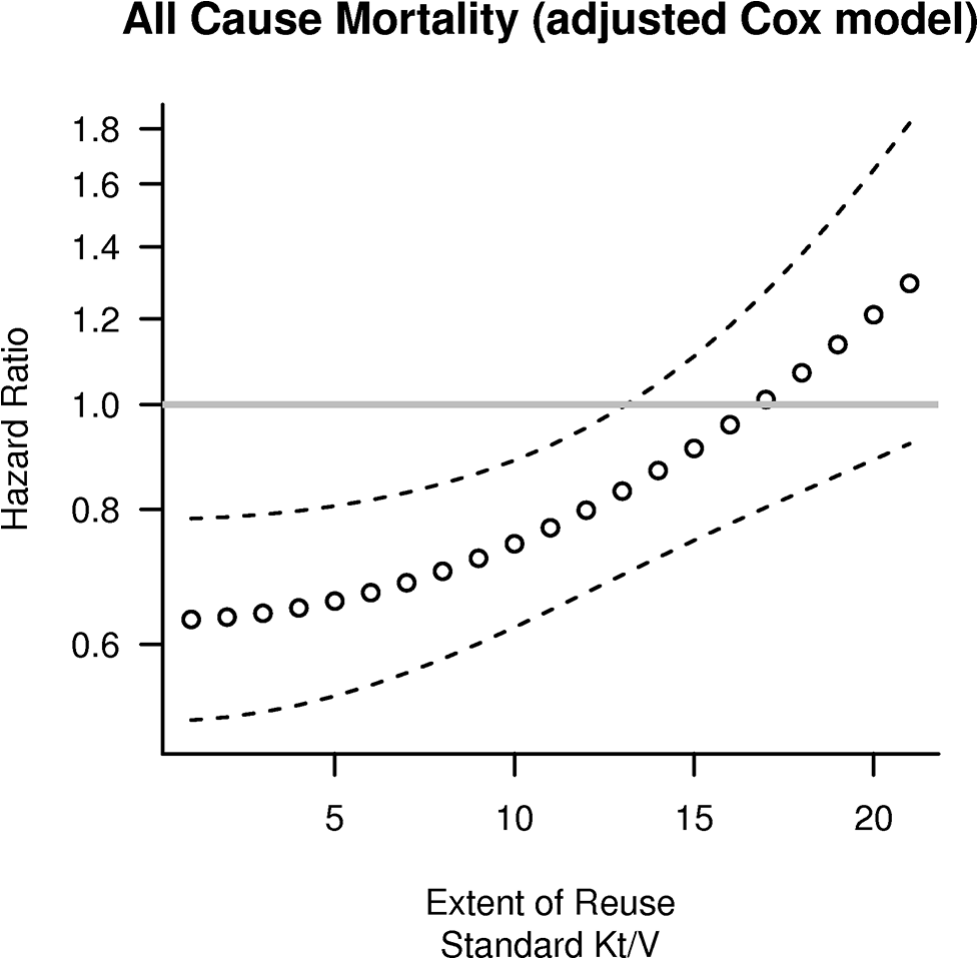
Predicted Hazard Ratio (HR) of High Flux(HF) dialysis as a function of membrane reuse. Predictions were obtained by fitting a meta-regression model to adjusted (for age, gender, diabetes, vascular access and comorbidity score) HR of subgroups of HEMO (defined on the basis of reuse quartiles, Kt/V and flux) and MPO. The meta-regression model accounted for Kt/V and the (square) of the extent of reuse. Of note, reuse of HF membranes up to 13 times appears to yield a protective effect with a predicted RR of 0.83, and an associated 95% CI: 0.70–0.99 that excludes unity.

**Fig 6:**
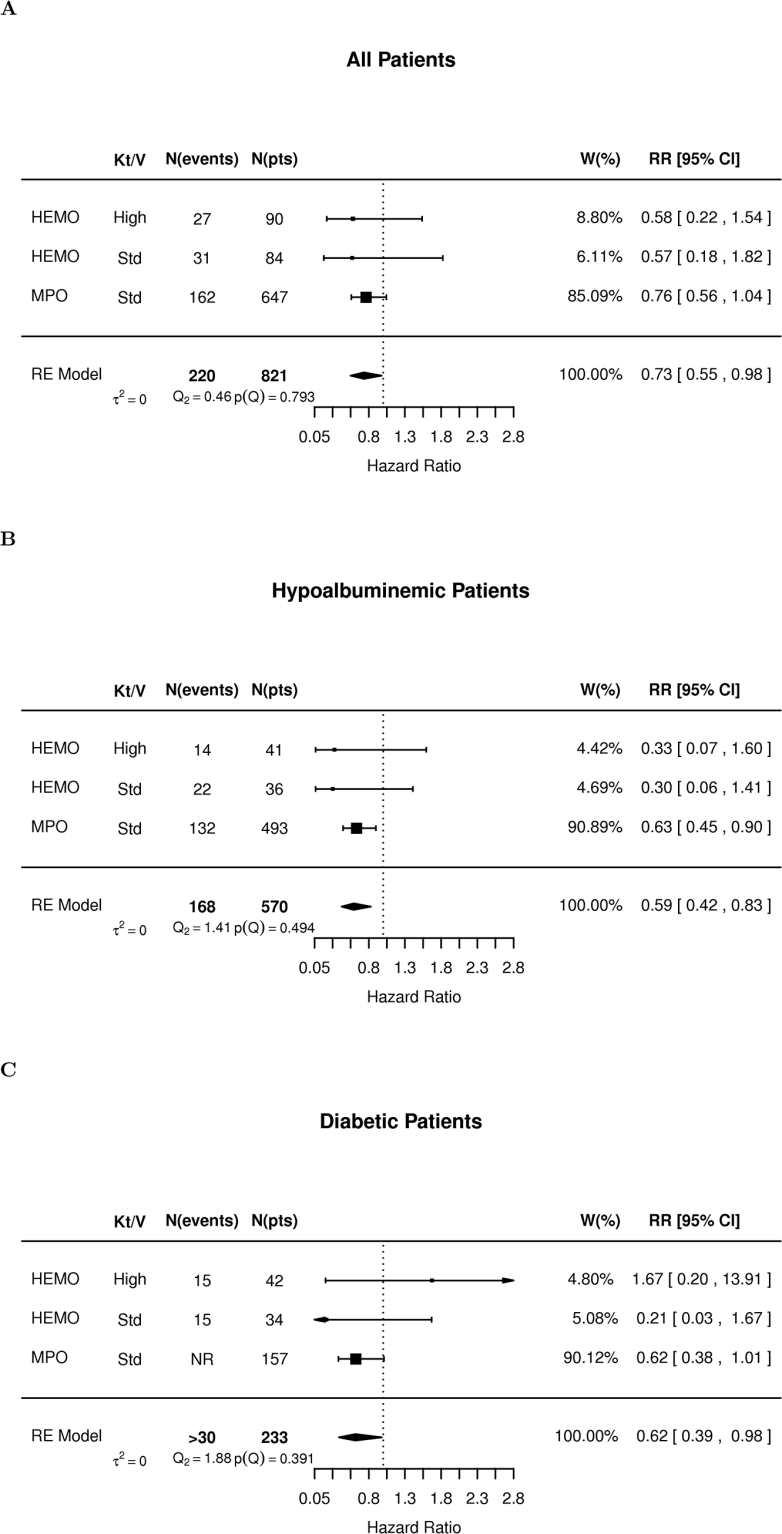
Meta-analysis of adjusted Hazard Ratio in HEMO study participants dialyzed with non-reused HF membranes and MPO patients. (A) all patients, (B) hypo-albuminemic patients, (C) patients with diabetes.

In meta-regression analyses, both linear and quadratic relationships between flux and number of reuses were examined, with the later providing better fit to the data (S1 Text: Supplementary Results). Model predictions for the HR of HF dialysis for different number of reuses and standard Kt/V dialysis dose are shown in Fig 5; the model predicts an adjusted HR of 0.63 (95% CI: 0.51–0.78) for HF dialysis with non-reused membranes, with the relative effects of flux progressively weaning after the 7^th^ reuse. When we examined outcomes in patients with hypo-albuminemia and in diabetics we obtained similar results: a model predicted HR of 0.56, (95% CI: 0.36–0.86, p = 0.009) and HR of 0.64 (95% CI: 0.46–0.89, p = 0.008) respectively associated with HF dialysis with non-reused dialyzers (graphs not shown). When we considered only the HEMO participants dialyzed with non-reused membranes, the results were consistent with the meta-regressions in showing a protective effect of HF dialysis that was numerically higher in hypo-albuminemic and diabetic patients (Fig 6).

## Discussion

In this secondary analysis of HEMO we found that minimally reused HF filters were associated with reduction in the hazard ratio for all-cause and cause-specific mortality relative to their LF counterparts, but this relation reversed at high levels of reuse. In joint modeling analyses of survival and predialysis β2M levels we found that this mortality pattern was not associated with differential effects on β2M concentration by reused dialyzers i.e. an indirect effect of flux. Evidence synthesis of the HEMO and MPO, the largest randomized trials to date to explore the effects of HF dialysis, support the beneficial effect of minimally reused HF dialyzers while suggesting a higher benefit for hypo-albuminemic patients and possibly for diabetics.

Our finding of a 25–30% relative risk reduction for patients dialyzed with minimally reused HF dialyzers appears plausible as it is consistent with multiple sources of data. Specifically, our numerical estimate is nearly equal to the effects of HF dialysis in MPO (in which reuse was not permitted) and is also in line with existing observational studies [21] that were used in the design of HEMO [8,25] and MPO [36]. Furthermore, the reduction in all-cause mortality is driven by reductions in cardiac and cardiovascular mortality. This pattern is consistent with multiple non-randomized analyses of the outcomes of HF dialysis [3,5–7,38]. In addition, the effects of HF dialysis appear larger in magnitude among hypo-albuminemic and diabetic patients in whom previous pre-specified RCT [10] and post-hoc [6] observational analyses have respectively suggested a larger benefit. Lastly, our meta-analysis of HEMO and MPO, which accounts for the differences in dialysis dose and reuse, shows a consistent benefit of non-reused and minimally reused HF dialyzers effects on all-cause mortality. Our simulation analyses utilizing the HEMO dataset predict that the adoption of HF dialysis in the US may have contributed to the 25% relative decline in mortality of the prevalent hemodialysis population seen over the last 15 years [49].

A tacit assumption made by investigators considering the overall null results of HEMO[10,50] is that reuse somehow render higher flux dialyzers less effective compared to their non-reused counterparts. Our analysis of the HEMO data does find an association between reuse and dialyzer flux, but in the opposite direction: rather than negatively affecting the survival of patients dialyzing with reused HF membranes, reuse improved the outcomes of LF dialysis. To our knowledge this is the first analysis to highlight the importance of the extent of reuse and membrane flux in addition to membrane material and reprocessing technique [51] on outcomes The existing literature includes harmful [51–54], protective [55] or null associations [13,56] and an overall neutral effect on mortality across the various studies [57]. The conflicting nature of the literature seems hardly surprising given the numerous factors that could confound the relation between reuse and outcomes. In particular, one could posit that reuse may be affecting RRF, an underappreciated but powerful predictor of survival in hemodialysis[24,26,27,58–60]. This hypothesis seems unlikely as an explanation for the associations we reported in HEMO, since the study enrolled patients with minimal RRF (urea clearance <0.5 ml/min) and most patients (67%) were anuric at baseline. Hence, an analysis of reuse on residual diuresis over time is limited by the small number of study participants that simultaneously had non-negligible urine output at baseline and follow up. Previous investigators have looked into the question of changes in RRF and beta 2 microglobulin in HEMO [61] and concluded that “*rigorous modeling the rate of decline in residual kidney function is prohibited because the majority of patients had no measurable residual kidney clearance at baseline*”.

To better understand the effects of HF dialysis on outcomes in the HEMO Study, we jointly modeled survival and β_2_M concentration in study participants. This novel analysis suggests that changes in β_2_M concentration explain one third of the estimated benefit of minimally reused HF dialyzers, while the remainder is accounted for by direct effects which are not related to β_2_M. Thus, in spite of the theoretical predominance of the “middle molecule” hypothesis on dialysis technology research[62], a closer examination of HEMO suggests that removal of these uremic solutes may have only a modest effect on outcomes. Although β_2_M is considered a marker for the entire spectrum of “middle” molecule toxins [63,64], other uremic retention solutes may demonstrate dialytic kinetics that differ from those of β_2_M [65,66]. It is conceivable that the direct flux effect, could be ascribed to an imprecise quantification of middle molecule exposure by the predialysis β_2_M concentration, the removal of other molecules or to non-clearance effects of dialyzers. Recent work by our group shows that the predialysis β_2_M concentration is an accurate measure of the time averaged concentration of β_2_M[67,68], so that the first hypothesis appears unlikely. Although research to date has largely focused on β_2_M kinetics [69] and its involvement in dialysis related amyloidosis, the knowledge gap in the field of uremic toxicity does not rule out the possibility i.e. the direct effect of flux is HEMO is mediated through the clearance of non-β_2_M “large” molecules. However, this appears a less likely explanation since the clearance of these molecules by HF dialyzers is smaller than the clearance of urea and β_2_M which were both controlled by HEMO. Consequently, the possibility that reused membranes exhibit differential kinetics of elimination is more theoretical than real. Even if differential kinetics of large molecules was they play, the latter would have to exhibit an improbable combination of high threshold for toxicity (otherwise patients would not survive long enough to be enrolled in HEMO) and an extremely adverse toxicity profile after the threshold (otherwise a differential effect would not be observed in HEMO). These considerations argue that non-clearance attributes of dialyzers explain the direct effects of flux and its modification by reuse.

An insight into the processes underlying the direct effects of flux and the effects of reuse comes from our dynamic outcomes simulations in which reuse had a striking modulating effect on LF but not on HF dialysis. Compared to non-reused LF membranes, reused ones were associated with improved survival, while reusing HF dialyzers had essentially no impact on survival. As dialyzer reuse [70] is generally [71–73], but not invariably [74–76] associated with enhanced biocompatibility and reduced oxidative stress, it would be tempting to speculate that this discrepancy can be attributed to an improvement in biocompatibility seen only with reuse of LF dialyzers. However, a selective increase in the biocompatibility of LF dialyzers by reuse appears unlikely for a number of reasons. Firstly, bioincompatible membranes were prohibited in HEMO [9,16] ruling out a difference in the ex-factory biocompatibility between HF and LF dialyzers. Furthermore, had a selective reuse-related improvement in biocompatibility occurred, one would expect this to be associated with reduced production rates of inflammatory biomarkers (including β_2_M [73]) only with LF dialysis [71]. In a study, such as HEMO, of functionally anephric patients in which middle molecule clearance is stably controlled by protocol, higher rates of β_2_M production would be associated with progressive accumulation over time. However reuse v.s non-reuse decreased the chronic slope in the β_2_M concentration to a similar extent in the two study arms. Therefore, if biocompatibility was improved by reuse, it probably did so to the same extent for LF and HF membranes. This conclusion is compatible with a previous report in a subgroup of patients from HEMO which showed that reuse improved phagocytic cell function (an index of membrane biocompatibility [77,78]) irrespective of membrane flux [79]. Taken together these considerations suggest that reuse improved biocompatibility of both LF and HF dialyzers in HEMO but additional factors have to be invoked to explain the lack of outcome improvement with reuse of HF dialyzers.

Previous assessments of HF dialyzers [80–84] by morphometric (via Atomic Force and Transmission Electron Microscopy), protein elution and immunoblotting methods have revealed alterations in morphology (increased pore size, cracking) and protein deposition in the dialysate side of the HF membranes associated with first use and subsequent reuses. These proteins with a molecular weight < 30kDa correspond to middle molecules that had been filtered in previous dialysis sessions with the same membrane and could (along with chemical residues from the fractured membrane material) theoretically been back-filtered to the patient during subsequent dialysis sessions. As such proteins are not filtered at all by LF membranes the combination of previous deposition in the internal (dialysate side), enhanced permeability and back filtration of previously filtered middle molecules could offer an attractive explanation of the differential effects of reuse on outcomes with HF and LF membranes.

The use of HF dialyzers is now widespread worldwide [2] and will increase further as convective therapies (hemodiafiltration/hemofiltration) that require these filters become established through evidence from randomized trials [85–87]. Our analysis of reuse in HEMO and the synthesis of HEMO and MPO have implications for nephrology practice. In the developed world, where reuse of dialyzers is either prohibited by law (European Union and Japan) or not practiced as a result of operational protocols (the majority of the dialysis providers in the USA), we suggest that LF membranes should be abandoned in favor of HF dialyzers. To the extent that HF dialyzer reuse is a necessary component of a dialysis program for environmental [57], financial (e.g. other dialysis providers in the USA) [88] or resource constraint (as in lower income countries) [14,76,89,90] reasons, we believe that reused HF dialyzers may realize the same benefits over non-reused LF dialyzers and thus HF membranes should also be preferred. Though extensive reuse of LF dialyzers is unlikely to ever be practiced except in severely resource-constrained settings, our re-exploration of HEMO suggests that there may be upsides in not adopting HF dialysis in these environments. Furthermore, in such systems substantial proportion of dialysis units lack proper water treatment facilities [14,89] making the use of HF membranes risky as a result of backfiltration [91,92], while the reduced (usually biweekly) dialysis frequency limits the cumulative removal of middle molecules.

These findings should be interpreted in light of the following limitations. First, our analysis is a secondary one, suitable for generating hypothesis for further studies. HEMO was not designed to test the effects of non-reused HF dialyzers, hence we cannot rule out the possibility of chance underlying the beneficial effects of non-reused to slightly reused HF dialyzers. Nevertheless, a large number of confounding factors, which could have conceivably affected outcomes assessment (including baseline urine production), did not differ between the two flux arms within reuse quartiles. Furthermore, a patient level meta-analysis between HEMO and MPO should be undertaken to corroborate the impression that the flux effect appears to be larger in patients with hypo-albuminemia and diabetes while examining the cardiovascular and infectious components of mortality. Such an analysis could also consider the residual renal function and its evolution over time in both studies in order to explore the combined dialytic/residual renal clearance on outcomes. Second, the reason for the apparent attenuation in relative survival of patients dialyzed with HF dialyzers cannot be discerned from this report. As this attenuation does not appear to be related to β2M concentration or clearance, analyzing the HEMO samples in the NIDDK biobank repository [93,94] could offer some insights about a putative biocompatibility benefit limited only to LF membranes or the backfiltration hypothesis we endorse.

As the largest study of dialyzer flux in chronic hemodialysis patients, the HEMO Study findings have strongly influenced patient care and are referenced by the US [95], UK Renal Association [96], European Best Practice Guidelines on Hemodialysis Strategies [97], and the Australian Hemodialysis Clinical Guidelines [98]. This work provides a more detailed examination of variability of outcomes related to dialyzer reuse in HEMO. Our analysis uncovers hitherto unrecognized compatibilities in the findings of HEMO and MPO with respect to the overall effects of non-reused HF dialyzers, but also in the clinically important subgroups of hypo-albuminemic and diabetic patients. As these two studies contribute the bulk of RCT evidence for or against higher permeability membranes, our analysis may allow for a more precise appraisal of the available body of data justifying the widespread use of HF dialyzers. Furthermore, this analysis reinforces the recent guideline updates [98,99] which endorse the use of HF membranes in clinical practice. Future examination of longitudinal changes in biomarkers in previously established bio-repositories and of physicochemical properties of membranes may be able to pinpoint the non-clearance factors that explain the effects of dialyzer flux. Finally such an examination will suggest novel ways to materialize technological innovations to further improve patient outcomes.

## Supporting Information

S1 Fig Results of JM in which reuse was treated as continuous, time updated variable. The longitudinal and survival sub models were adjusted for the same variables as in Table 4; the panels (in clockwise order) show the relative reduction in β2M six months after the beginning of the study and the corresponding indirect, direct and total effects of HF dialysis as a function of the cumulative number of reuses. The relative reduction in predialysis β2M concentration was only minimally affected by reuse, while the direct and consequently the total effect of HF dialysis was related to the extent of reuse. Relative to LF dialyzers, HF membranes were associated with reduced hazard ratio of death as long as they were reused for less than 8 times, while reuse of HF dialyzers for more than 17 times was associated with relative increases in mortality (HR>1).
(TIFF)

S2 Fig Meta-analysis of adjusted (for diabetes, vascular access, comorbidity score, age, gender) HR of HF dialysis between HEMO and MPO. In the meta-analysis of HEMO and MPO a substantial amount of heterogeneity across the range of membrane reuse and small molecule clearance used in the two studies was noted: Q = 20.4, p(Q) = 0.009. (A) all patients, (B) hypoalbuminemic patients, (C) patients with diabetes.
(TIFF)

S1 Table Baseline Characteristics of HEMO participants in the lowest reuse quartile (DOC)

S2 Table Baseline Characteristics of HEMO participants in the second reuse quartile (DOC)

S3 Table Baseline Characteristics of HEMO participants in the third reuse quartile (DOC)

S4 Table Baseline Characteristics of HEMO participants in the highest reuse quartile (DOC)

S5 Table Dialyzer Reuse Method Combinations used in HEMO participants (DOC)

S6 Table Survival Analysis in HEMO with Cox models incorporating reuse by flux interactions (DOC)

S7 Table Hazard Ratio of Death between HF and LF dialysis versus extent of reuse adjusting for time of randomization. In these models, the cumulative mean number of reuses (available monthly for each patient) was used.
(DOC)

S1 Text Supplementary Methods and Results (DOC)
