# Supplementary figures and images for: Dialyzer Reuse and Outcomes of High Flux Dialysis
Source: PLoS One. 2015 Jun 9;10(6):e0129575. doi: 10.1371/journal.pone.0129575 (PMC4461247; doi:10.1371/journal.pone.0129575)

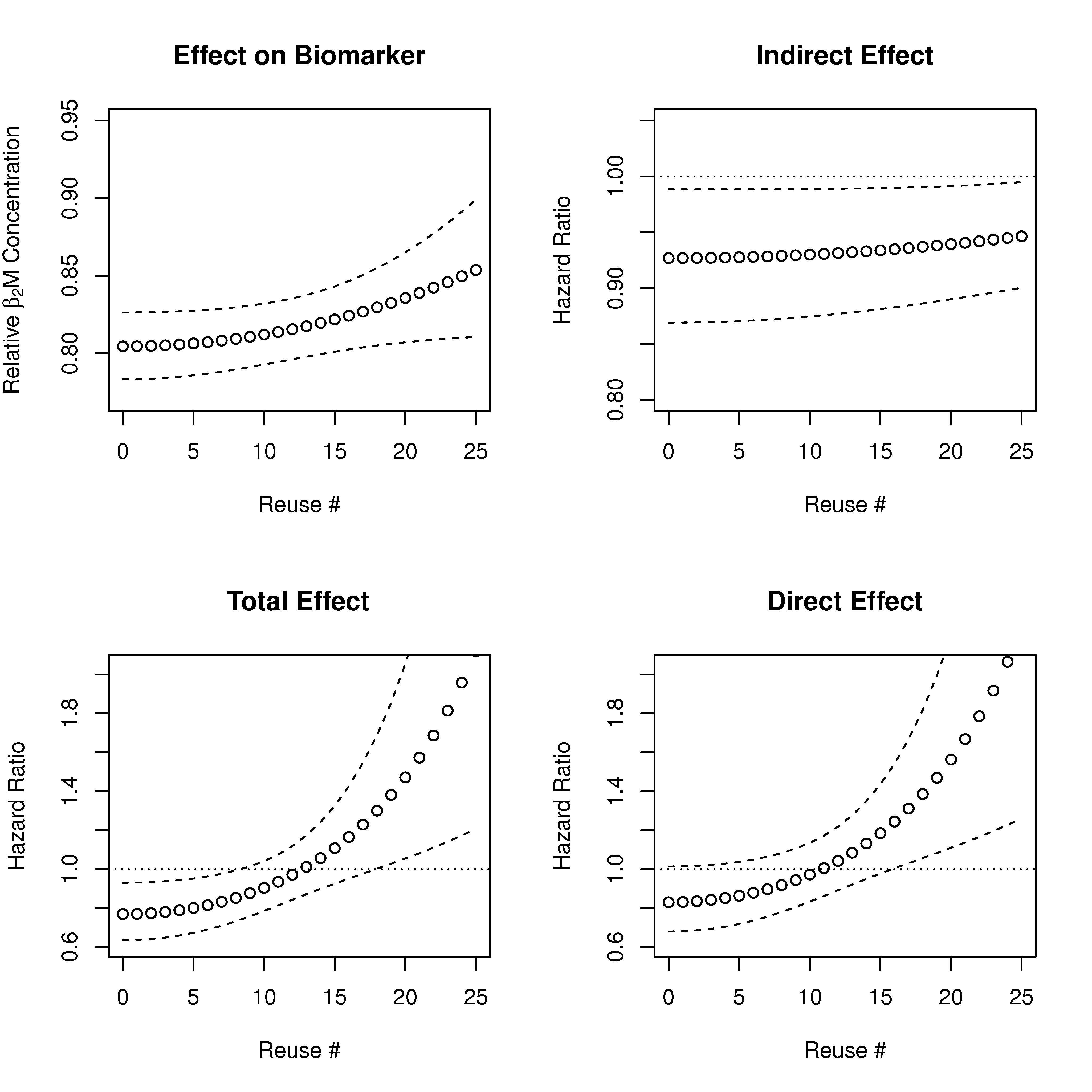

Supplement: S1 Fig — The longitudinal and survival sub models were adjusted for the same variables as in Table 4; the panels (in clockwise order) show the relative reduction in β2M six months after the beginning of the study and the corresponding indirect, direct and total effects of HF dialysis as a function of the cumulative number of reuses. The relative reduction in predialysis β2M concentration was only minimally affected by reuse, while the direct and consequently the total effect of HF dialysis was related to the extent of reuse. Relative to LF dialyzers, HF membranes were associated with reduced hazard ratio of death as long as they were reused for less than 8 times, while reuse of HF dialyzers for more than 17 times was associated with relative increases in mortality (HR>1). (TIFF) [file pone.0129575.s001.TIFF]

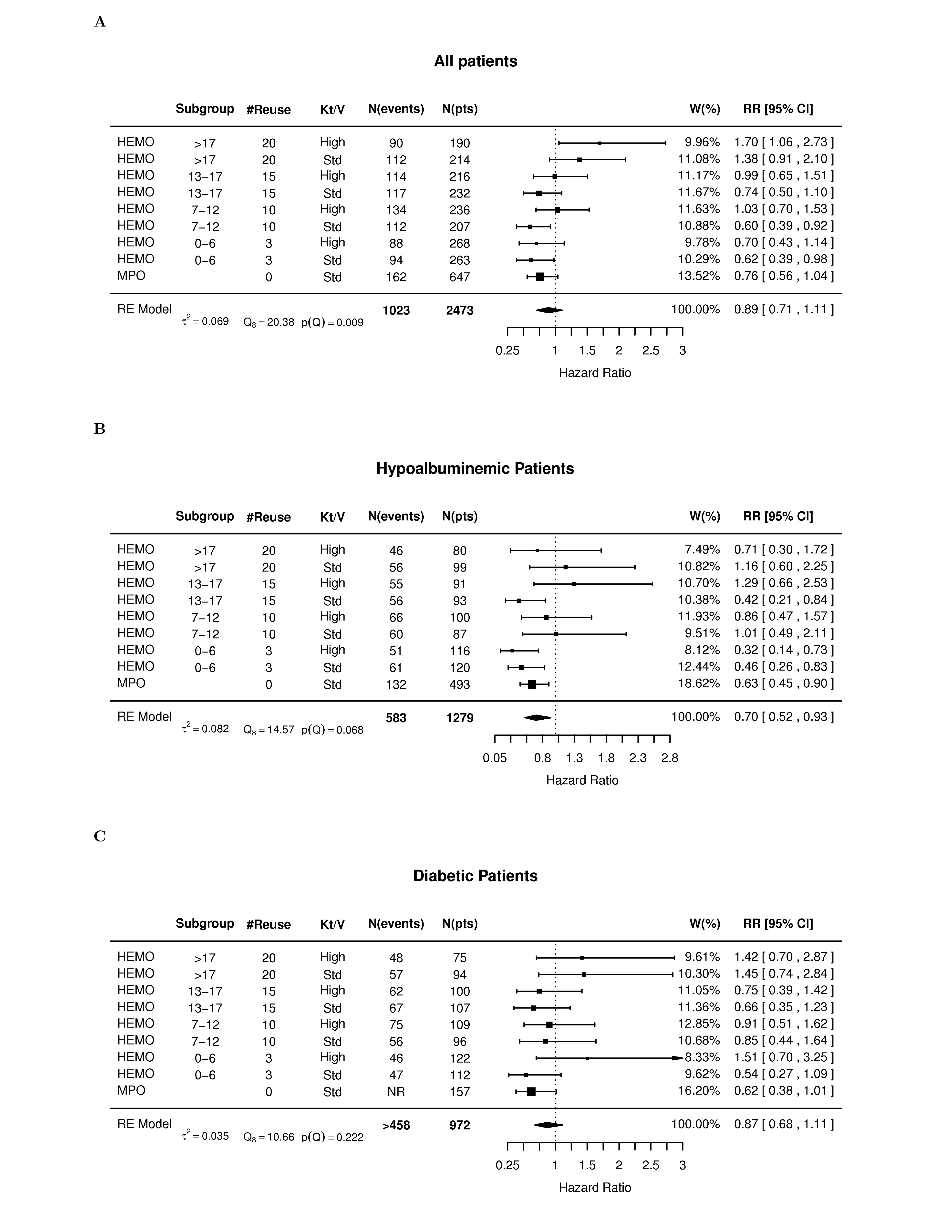

Supplement: S2 Fig — In the meta-analysis of HEMO and MPO a substantial amount of heterogeneity across the range of membrane reuse and small molecule clearance used in the two studies was noted: Q = 20.4, p(Q) = 0.009. (A) all patients, (B) hypoalbuminemic patients, (C) patients with diabetes. (TIFF) [file pone.0129575.s002.TIFF]
