# Supplementary material for: Dialyzer Reuse and Outcomes of High Flux Dialysis
Source: PLoS One. 2015 Jun 9;10(6):e0129575. doi: 10.1371/journal.pone.0129575 (PMC4461247; doi:10.1371/journal.pone.0129575)
Supplement: S2 Table — (DOC) [file pone.0129575.s004.doc]

Table S2 Baseline Characteristics of HEMO participants in the second reuse quartile

|  | **Low Flux** | **High Flux** | **p** |
| --- | --- | --- | --- |
| **N** | 230 | 213 |  |
| **Kt/V Assignment** |  |  | 0.77 |
| High Kt/V | 53% (121) | 54% (115) |  |
| **Pre-randomization membrane Flux** |  |  | 0.63 |
| High Flux | 64% (117) | 67% (142) |  |
| **Age** | 57.5 ± 14.5 | 58.0 ± 12.8 | 0.96 |
| **Race** |  |  | 0.23 |
| Black | 70% (161) | 75% (160) |  |
| **Diabetic Status** |  |  | 0.77 |
| Diabetic | 47% (108) | 46% (97) |  |
| **Gender** |  |  | 0.88 |
| Female | 56% (128) | 56% (120) |  |
| **Duration (years)** | 3.92 ± 4.50 | 3.81 ± 4.37 | 0.83 |
| **Cause of ESRD** |  |  | 0.61 |
| Glomerulonephritis | 16% (36) | 14% (30) |  |
| Hypertension | 30% (70) | 33% (70) |  |
| Diabetes | 38% (88) | 37% (79) |  |
| Ischemic Nephropathy | 5% (11) | 5% (10) |  |
| Acute Renal Disease | 2% (4) | 4% (8) |  |
| Other | 9% (21) | 7% (16) |  |
| **Albumin (g/dl)** | 3.62 ± 0.36 | 3.65 ± 0.35 | 0.31 |
| **Residual Renal Function** |  |  | 0.30 |
| Urine Volume (>200 ml/day) | 8% (18) | 11% (23) |  |
| **ICED** | 1.93 ± 0.84 | 1.98 ± 0.83 | 0.26 |
| **Vascular Access** |  |  | 0.73 |
| Arteriovenous Fistula | 34% (78) | 33% (70) |  |
| Arteriovenous Graft | 58% (133) | 59% (125) |  |
| Catheter/Other | 0% (1) | 1% (3) |  |
| **Year of Randomization**  (since the beginning of HEMO) | 1.69 ± 1.44 | 1.69 ± 1.38 | 0.84 |

Summary statistics are presented as mean ± standard deviation and as % (frequencies) for continuous and categorical variables respectively. Tests used to compare variables Pearson test (categorical), Kruskal-Wallis (continuous). The total number of patients (1826) differs from the number of patients enrolled in HEMO, because there was no available information about reuse for 20 subjects who died before their first post-enrolment evaluation. There was no difference in the distribution of patients randomized to either LF or HF membranes among the fifteen participating centers (p=0.11)
