# Supplementary material for: Dialyzer Reuse and Outcomes of High Flux Dialysis
Source: PLoS One. 2015 Jun 9;10(6):e0129575. doi: 10.1371/journal.pone.0129575 (PMC4461247; doi:10.1371/journal.pone.0129575)
Supplement: S5 Table — (DOC) [file pone.0129575.s007.doc]

|  | **High Flux** | | | | **Low Flux** |
| --- | --- | --- | --- | --- | --- |
| **Dialyzer** | *CT 190G* | *F80A* | *F80B* | *Other* | *All Types* |
|  | (N=15622) | (N=7190) | (N=6846) | (N=2368) | (N=30358) |
| **Reuse Method**  **(Bleach/Disinfectant)** |  |  |  |  |  |
| No-Bleach / None | 22% (3405) | 14% (1031) | 11% (728) | 76% (1790) | 24% (7387) |
| Bleach / None | 0% (1) | 0% (1) | 0% (1) | 0% (1) | 0% (1) |
| No-Bleach / Formaldehyde | 0% (5) | 0% (5) | 0% (6) | 0% (0) | 0% (42) |
| Bleach / Formaldehyde | 10% (1501) | 8% (592) | 60% (4112) | 3% (69) | 20% (6011) |
| No-Bleach/ Gluteraldehyde | 1% (154) | 0% (2) | 1% (39) | 1% (29) | 1% (200) |
| Bleach / Gluteraldehyde | 10% (1598) | 1% (48) | 7% (459) | 10% (237) | 8% (2455) |
| No-Bleach / Renalin | 57% (8941) | 46% (3328) | 2% (110) | 8% (190) | 36% (10883) |
| Bleach / Renalin | 0% (16) | 0% (8) | 20% (1391) | 1% (33) | 5% (1593) |
| No-Bleach / Heated Citric Acid | 0% (1) | 30% (2170) | 0% (0) | 1% (19) | 6% (1783) |
| Bleach / Heat Citric Acid | 0% (0) | 0% (5) | 0% (0) | 0% (0) | 0% (3) |
