# Supplementary material for: Dialyzer Reuse and Outcomes of High Flux Dialysis
Source: PLoS One. 2015 Jun 9;10(6):e0129575. doi: 10.1371/journal.pone.0129575 (PMC4461247; doi:10.1371/journal.pone.0129575)
Supplement: S6 Table — (DOC) [file pone.0129575.s008.doc]

Table S6 Survival Analysis in HEMO with Cox models incorporating reuse by flux interactions

|  | **All Cause Mortality‖** | **Cardiac Mortality‡** | **Cardiovascular Mortality†** | **Infectious Mortality•** |
| --- | --- | --- | --- | --- |
| **Main Effect of Flux (HF)** | 0.67 (0.48 – 0.92)  p=0.015 | 0.64 (0.44 – 0.95)  p=0.03 | 0.61 (0.41 – 0.90)  p=0.012 | 0.53 (0.28 – 1.02)  p=0.057 |
| **Main Effect of Reuse** |  |  |  |  |
| Second Reuse Quartile (Q2) | 0.72 (0.39 – 1.33)  p=0.29 | 1.00 (0.44 – 2.30)  p=0.98 | 0.96 (0.42 – 2.17) p=0.93 | 0.25 (0.08 – 0.77) p=0.016 |
| Third Reuse Quartile (Q3) | 0.58 (0.30 – 1.13)  p=0.11 | 0.58 (0.22 – 1.49) p=0.26 | 0.55 (0.23 – 1.33) p=0.18 | 0.39 (0.11 – 1.43) p=0.16 |
| Fourth Reuse Quartile (Q4) | 0.21 (0.10 – 0.44)  p<0.001 | 0.35 (0.16 – 0.78) p=0.01 | 0.27 (0.13 – 0.55) p<0.001 | 0.12 (0.03 – 0.44) p=0.001 |
| **Interaction between Reuse and Flux** |  |  |  |  |
| HF x Q2 | 1.29 (0.82 – 2.03)  p=0.27 | 1.06 (0.59 – 1.92) p=0.83 | 1.02 (0.56 – 1.85) p=0.95 | 2.08 (0.98-4.45) p=0.057 |
| HF x Q3 | 1.28 (0.82 – 1.99)  p=0.26 | 1.28 (0.71 – 2.31) p=0.41 | 1.33 (0.74 – 2.39) p=0.33 | 1.20 (0.48 – 3.01) p=0.69 |
| HF x Q4 | 2.18 (1.41 – 3.37)  p<0.001 | 1.52 (0.90 – 2.58) p=0.11 | 1.90 (1.20 – 3.01) p=0.006 | 2.44 (1.12 – 5.30) p=0.024 |

In these regressions, the referent (baseline risk) category is that of patients dialyzing with minimally reused, LF dialyzers. The main effect of flux corresponds to the relative risk (RR) of patients dialyzing with minimally reused HF dialyzers v.s. the referent. The main effects of reuse correspond to the RR of patients exposed to reused LF dialyzers v.s. the baseline category, while the interactions between reuse quartile and flux quantify the modifying effect of reuse on outcomes of HF dialysis. To predict the RR of moderately-extensively reused HF dialyzers (2^nd^-4^th^ reuse quartiles) against the minimally reused HF ones, one multiplies the main effects of reuse by the corresponding interaction term. The predicted RR of reused HF dialyzers against their reused LF counterparts at a given reuse quartile is given by the product of the main effect of flux by the interaction term between the given quartile and flux.

RR estimates were obtained from a Cox regression model adjusting for reuse quartile, Flux and Kt/V assignments, age, sex, diabetes, duration of ESRD dependency (upon study enrolment), ICED, albumin, vascular access, pre-randomization flux, residual urine volume, and finally the interaction between reuse quartile and flux; the baseline hazard was stratified by study center. Reported Relative Risks are model predictions and associated 95% CI for patients dialyzing with HF relative to patients dialyzing with LF ones, reused to the same extent as the former.

**P (linear test for trend): ‖ p <0.001 for both the main effect of reuse and the interaction between reuse and flux, ‡ p 0.006 for the main effect of reuse and p=0.10 the interaction between reuse and flux, † p<0.001 for the main effect of reuse and p=0.005 for the interaction between reuse and flux, • p=0.003 for the main effect of reuse and p=0.08 for the interaction between reuse and flux**
