# Supplementary material for: Dialyzer Reuse and Outcomes of High Flux Dialysis
Source: PLoS One. 2015 Jun 9;10(6):e0129575. doi: 10.1371/journal.pone.0129575 (PMC4461247; doi:10.1371/journal.pone.0129575)
Supplement: S7 Table — In these models, the cumulative mean number of reuses (available monthly for each patient) was used. (DOC) [file pone.0129575.s009.doc]

**Table S7** Relative Risks of Death between HF and LF dialysis versus extent of reuse adjusting for time of randomization

|  | **1^st^ Quartile** | **2^nd^ Quartile** | **3^rd^ Quartile** | **4^th^ Quartile** |
| --- | --- | --- | --- | --- |
| **All Cause Mortality** | 0.67 (0.48 – 0.93)  p=0.017 | 0.85 (0.65 – 1.12) p=0.26 | 0.86 (0.65 – 1.14)  p=0.31 | 1.45 (1.13 – 1.86) p=0.004 |
| **Cardiac Mortality** | 0.67 (0.43 – 0.96)  p=0.03 | 0.67 (0.42 – 1.05)  p=0.082 | 0.84 (0.55 – 1.29)  p=0.43 | 0.98 (0.68 – 1.42)  p=0.93 |
| **Cardiovascular Mortality** | 0.61 (0.41 – 0.90)  p=0.01 | 0.61 (0.39 – 0.94)  p=0.02 | 0.82 (0.55 – 1.22) p=0.33 | 1.16 (0.87 – 1.53) p=0.32 |
| **Infectious Mortality** | 0.54 (0.28 – 1.05)  p=0.07 | 1.12 (0.68 – 1.85)  p=0.66 | 0.67 (0.37 – 1.24)  p=0.20 | 1.28 (0.81 – 2.01)  p=0.29 |

Relative Risk (RR) estimates were obtained from a time updated Cox regression model adjusting for quartiles of cumulative reuse (updated for changes in reuse during the study), Kt/V and Flux assignments, the interaction between reuse quartile and flux, baseline covariates: age, sex, diabetes, duration of ESRD dependency, ICED, albumin, vascular access and stratified by study center. Reported Relative Risks are model predictions and associated 95% CI for patients dialyzing with HF relative to patients dialyzing with LF ones, reused to the same extent as the former.
