# Supplementary material for: Dialyzer Reuse and Outcomes of High Flux Dialysis
Source: PLoS One. 2015 Jun 9;10(6):e0129575. doi: 10.1371/journal.pone.0129575 (PMC4461247; doi:10.1371/journal.pone.0129575)
Supplement: S1 Text — (DOC) [file pone.0129575.s010.doc]

**Supplementary Methods**

*Reuse Information in the Joint Model*

Patients were classified to reuse quartiles as stated in the main text and the interaction between reuse quartile and flux assignment was specified in both the survival and the longitudinal/repeated measures component of the joint model. Using these models we tested the modifying effects of reuse on flux assignment upon the indirect effects of flux (α) and the trajectory of β2M (β). In sensitivity analyses we tested the interaction between reuse, flux and their combination on the estimated effect of β2M on survival (γ). **These models, explore the hypothesis that a given β2M concentration may have a quantitatively different effect on survival in patients dialyzing with membranes of different flux and/or extent of reuse.** Finally, we evaluated the robustness of the reported findings by including the cumulative mean number of reuses as a time updated covariate in the JM. Thus, these JM incorporated two time updated covariates: predialysis β2M concentration (an internal covariate analyzed by the JM) and reuse, an exogenous covariate determined by the study protocol and dialysis clinic practices. We analyzed the β2M in both the untransformed and the logarithmically transformed scale using the same model for the trajectory and interaction terms.

*Random Effects Structure in the Joint Model*

In the JM developed, we allowed each individual to have their own chronic slope term and different starting points according to reuse quartile (random slope and intercept model). Due to the large number of random effects implied by an unrestricted covariance matrix, a block diagonal form was assumed for the latter and thus five random effects were fitted to the full dataset. In models in which the cumulative mean number of reuses appear, an unstructured covariance matrix was used in order to minimize assumptions made.

*Reporting of JM results*

To report the results of the JM, we graphed the distribution (mean and standard deviation) of the patient-level predicted β2M at the location of the knots, and also at 1, 3 and 6 years after randomization. The effects of reuse and flux on β2M during the two phases of the β2M trajectory were assessed on the average slope from the beginning of the study to 180 days and the chronic slope respectively. The magnitude, standard errors and the associated p-values of both these slopes were computed from the relevant linear transformation of the estimated natural spline coefficients and the associated variance-covariance matrix produced by the JM fitting procedure.

As the JM treats β2M as a time dependent covariate, the relative risk associated with higher levels of β2M is a function of time so that the associated hazard rate ration is also time-varying. To facilitate comparisons between the β2M-associated survival effects of different flux and reuse combinations we computed relative risk ratios (direct and indirect) at the end of the acute phase (6-months), since most temporal changes in β2M and risk were much less pronounced after this point.

*Testing interactions between reuse and flux*

In all regression models (survival, linear mixed or joint) incorporating interactions between flux and reuse variables, we report the statistical significance of the interaction term appearing in the model (Wald test). Reuse effects are presented as model predictions (and the associated 95% CI) about the impact of HF membranes relative to their LF counterparts subjected to the same extent of reuse. In JMs examining the functional relationship between the cumulative number of reuses and outcomes, we applied informational theory criteria (Akaike Information Criterion) to select the best fitting model when reporting results.

*JM based dynamic Simulations of Survival under Different Extents of Reuse and Membrane Flux*

In order to illustrate the impact of adopting different reuse strategies for either LF or HF dialyzers on outcomes, we utilized the capability of Joint Models to yield dynamic, subject specific survival probabilities from a baseline set of measurements. Briefly, we took the fitted joint models incorporating baseline covariates, the first available set of β2M measurements for each patient and the study membrane flux and assigned each of these “simulated” patients to all possible combinations of reuse and flux. Subsequently, we used the JM to dynamically simulate the outcomes under the eight possible reuse/flux combinations over the time horizon of these hypothetical trials. Based on these simulations we generated survival curves for each individual at each time point. In this context, survival estimates can be taken as indices of the causal treatment effect of flux and reuse for each particular patient. By averaging these estimates over all the individuals in the simulation studies, we obtained the average causal effect which was then used as the basis of comparative effectiveness assessments described in the paper. Differences in P5 between each particular flux/reuse combination and all the others were tabulated, and the uncertainty in these estimates was quantified by averaging over the samples obtained by techniques of Markov Chain Monte Carlo (MCMC) integration ([17,18]). As these are Bayesian estimates which do not admit a conventional p-value calculation for their comparison, we adopted the Bayesian literature convention of considering such differences “significant” if the corresponding 95% Credible Interval (CrI, the range of values in which 95% of the MCMC samples are contained) excludes zero.

### Meta-analysis and Meta-regression of HEMO and MPO

The dialysis interventions differed in the two trials not only with respect to reuse of membranes, but also with respect to the small molecule dose with the MPO intervention delivering a spKt/V very similar the standard spKt/V target in HEMO. Furthermore, there were major differences in patient demographics (prevalent patients in HEMO, incident dialysis patients in MPO), type of access and enrollment criteria (patients in HEMO had to have minimal residual renal function, while MPO included a stratum of hypoalbuminemic patients). Finally, the investigators used different statistical models to adjust survival in the primary reports of the two trials. To account for the differences in interventions, patients in HEMO were cross-classified according to Kt/V arm, flux and quartiles of reuse before computing discrete outcomes (number of events and patient years). To *align the statistical models used in the trials*, we re-analyzed survival in HEMO using the same statistical model that the MPO investigators used in their report. This model which adjusted outcomes for age, Kt/V, gender, diabetes, comorbidity score (ICED in HEMO, Charlson in MPO) and type of access yielded estimates for the Hazard Ratio within each of the eight cohorts in HEMO defined on the basis of reuse quartile and Kt/V assignment. The corresponding information was extracted from the MPO publication (Table 4 in [19]). To combine the evidence from both studies we utilized random effects meta-regression techniques adjusting for flux, spKt/V (coded as standard or high) and average reuse number of the patients in each HEMO reuse quartile. Heterogeneity in these analyses was quantified by the value of τ^2^ and assess by the Q statistic. In the meta-regression models we also explored non-linear relationships between reuse exposure and the effects of flux (linear v.s. quadratic), by comparing models with the Akaike Information Criterion (AIC). A sensitivity analysis involving only the HEMO patients who were dialyzed with non-reused membranes and the MPO study was also undertaken with conventional random effects meta-analysis methods.

We undertook subgroup analysis to examine the effects of flux on diabetic and hypo-albuminemic patients at baseline (defined as albumin < 4 g/dl in MPO). Hypo-albuminemia in HEMO was defined as having an immunonephelometry albumin of < 3.56 g/dl; this cutoff was based on previous research showing an average bias of 0.44 g/dl between Bromocresol Purple (the method commonly used by clinical laboratories which appeared to have been used in MPO) and immunonephelometry[4] (the method used to measure serum albumin in the HEMO trial).

## Supplementary Results

### Meta-analysis and Meta-Regression between HEMO and MPO

In the meta-analysis of HEMO and MPO we noted a substantial amount of heterogeneity across the range of membrane reuse and small molecule clearance used in the two studies : Q=20.4, p(Q)=0.009. Adjusting for either Kt/V or extent of reuse reduced heterogeneity, but the reduction was much larger with the reuse adjustment (Q=7.11, p(Q) =0.42 vs. Q=16.2, p(Q)=0.023 for Kt/V). A quadratic function of the extent of the reuse appears to yield better fitting meta-regression models on the basis of AIC (Akaike Information Criterion): 1.85 (quadratic) vs. 5.02 (linear) and was used in the final meta-regression model that accounted for both Kt/V and reuse. This model, which had the smallest heterogeneity (Q=4.9, p(Q)=0.55), estimated a statistically significant effect for HF dialysis (log-RR: -0.46, 95% CI: -0.68 – -0.24, p<0.001), modified by the square of the reuse number (log-RR: 0.0016, 95% CI: 0.0007 – 0.0026, p<0.001) and a non statistically significant effect of high Kt/V (log-RR: 0.21, 95% CI: -0.069-0.50, p=0.14).

**References**

1. Athienites NV, Miskulin DC, Fernandez GF, Bunnapradist S, Simon G, et al. (2000) Comorbidity assessment in hemodialysis and peritoneal dialysis using the Index of Coexistent Disease (ICED). Semin Dial 13: 320-326.

2. Miskulin DC, Athienites NV, Yan G, Martin AA, Ornt DB, et al. (2001) Comorbidity assessment using the Index of Coexistent Diseases in a multicenter clinical trial. Kidney Int 60: 1498-1510.

3. Carfray A, Patel K, Whitaker P, Garrick P, Griffiths GJ, et al. (2000) Albumin as an outcome measure in haemodialysis in patients: the effect of variation in assay method. Nephrol Dial Transplant 15: 1819-1822.

4. Clase CM, St Pierre MW, Churchill DN (2001) Conversion between bromcresol green- and bromcresol purple-measured albumin in renal disease. Nephrol Dial Transplant 16: 1925-1929.

5. Wells FE, Addison GM, Postlethwaite RJ (1985) Albumin analysis in serum of haemodialysis patients: discrepancies between bromocresol purple, bromocresol green and electroimmunoassay. Ann Clin Biochem 22 ( Pt 3): 304-309.

6. Leypoldt JK, Cheung AK, Deeter RB (1997) Single compartment models for evaluating beta 2-microglobulin clearance during hemodialysis. Asaio J 43: 904-909.

7. Cheung AK, Agodoa LY, Daugirdas JT, Depner TA, Gotch FA, et al. (1999) Effects of hemodialyzer reuse on clearances of urea and beta2-microglobulin. The Hemodialysis (HEMO) Study Group. J Am Soc Nephrol 10: 117-127.

8. Eknoyan G, Beck GJ, Cheung AK, Daugirdas JT, Greene T, et al. (2002) Effect of dialysis dose and membrane flux in maintenance hemodialysis. N Engl J Med 347: 2010-2019.

9. Santoro A, Mancini E, Bolzani R, Boggi R, Cagnoli L, et al. (2008) The effect of on-line high-flux hemofiltration versus low-flux hemodialysis on mortality in chronic kidney failure: a small randomized controlled trial. Am J Kidney Dis 52: 507-518.

10. Mandolfo S, Borlandelli S, Imbasciati E (2006) Leptin and beta2-microglobulin kinetics with three different dialysis modalities. Int J Artif Organs 29: 949-955.

11. Pellicano R, Polkinghorne KR, Kerr PG (2008) Reduction in beta2-microglobulin with super-flux versus high-flux dialysis membranes: results of a 6-week, randomized, double-blind, crossover trial. Am J Kidney Dis 52: 93-101.

12. Lonnemann G, Bahlmann FH, Freise J, Hertel B, Dinarello CA (2009) Low-flux hemodialysis suppresses interferon-gamma production: the possible role of beta2-microglobulin. Clin Nephrol 72: 170-176.

13. Mayer G, Thum J, Woloszczuk W, Graf H (1988) Beta-2-microglobulin in hemodialysis patients. Effects of different dialyzers and different dialysis procedures. Am J Nephrol 8: 280-284.

14. Ward RA, Greene T, Hartmann B, Samtleben W (2006) Resistance to intercompartmental mass transfer limits beta2-microglobulin removal by post-dilution hemodiafiltration. Kidney Int 69: 1431-1437.

15. Kanamori T, Sakai K (1995) An estimate of beta 2-microglobulin deposition rate in uremic patients on hemodialysis using a mathematical kinetic model. Kidney Int 47: 1453-1457.

16. Clark WR, Leypoldt JK, Henderson LW, Mueller BA, Scott MK, et al. (1999) Quantifying the effect of changes in the hemodialysis prescription on effective solute removal with a mathematical model. J Am Soc Nephrol 10: 601-609.

17. Rizopoulos D (2011) Dynamic predictions and prospective accuracy in joint models for longitudinal and time-to-event data. Biometrics 67: 819-829.

18. Rizopoulos D (2012) Joint Models for Longitudinal and Time-to-Event Data: With Applications in R; Chow S-C, editor: CRC Press. 275 p.

19. Locatelli F, Martin-Malo A, Hannedouche T, Loureiro A, Papadimitriou M, et al. (2009) Effect of membrane permeability on survival of hemodialysis patients. J Am Soc Nephrol 20: 645-654.
